# Preload-based Starling-like control of rotary blood pumps: An *in-vitro* evaluation

**DOI:** 10.1371/journal.pone.0172393

**Published:** 2017-02-17

**Authors:** Mahdi Mansouri, Shaun D. Gregory, Robert F. Salamonsen, Nigel H. Lovell, Michael C. Stevens, Jo P. Pauls, Rini Akmeliawati, Einly Lim

**Affiliations:** 1 Department of Biomedical Engineering, University of Malaya, Kuala Lumpur, Malaysia; 2 Innovative Cardiovascular Engineering and Technology Laboratory, Critical Care Research Group, the Prince Charles Hospital, Brisbane, Queensland, Australia; 3 School of Medicine, University of Queensland, Brisbane, Queensland, Australia; 4 School of Engineering, Griffith University, Brisbane, Queensland, Australia; 5 Department of Intensive Care, Alfred Hospital, Prahran, Victoria, Australia; 6 Department of Epidemiology and Preventive Medicine, Monash University, Melbourne, Victoria, Australia; 7 Graduate School of Biomedical Engineering, UNSW, Sydney, New South Wales, Australia; 8 School of Medicine, University of Sydney, Camperdown, New South Wales, Australia; 9 Department of Mechatronics Engineering, International Islamic University Malaysia, Kuala Lumpur; University of Adelaide, AUSTRALIA

## Abstract

Due to a shortage of donor hearts, rotary left ventricular assist devices (LVADs) are used to provide mechanical circulatory support. To address the preload insensitivity of the constant speed controller (CSC) used in conventional LVADs, we developed a preload-based Starling-like controller (SLC). The SLC emulates the Starling law of the heart to maintain mean pump flow ($\bar{{\text{Q}}_{\text{P}}}$) with respect to mean left ventricular end diastolic pressure (PLVED_m_) as the feedback signal. The SLC and CSC were compared using a mock circulation loop to assess their capacity to increase cardiac output during mild exercise while avoiding ventricular suction (marked by a negative PLVED_m_) and maintaining circulatory stability during blood loss and severe reductions in left ventricular contractility (LVC). The root mean squared hemodynamic deviation (RMSHD) metric was used to assess the clinical acceptability of each controller based on pre-defined hemodynamic limits. We also compared the in-silico results from our previously published paper with our *in-vitro* outcomes. In the exercise simulation, the SLC increased $\bar{{\text{Q}}_{\text{P}}}$ by 37%, compared to only 17% with the CSC. During blood loss, the SLC maintained a better safety margin against left ventricular suction with PLVED_m_ of 2.7 mmHg compared to -0.1 mmHg for CSC. A transition to reduced LVC resulted in decreased mean arterial pressure (MAP) and $\bar{{\text{Q}}_{\text{P}}}$ with CSC, whilst the SLC maintained MAP and $\bar{{\text{Q}}_{\text{P}}}$. The results were associated with a much lower RMSHD value with SLC (70.3%) compared to CSC (225.5%), demonstrating improved capacity of the SLC to compensate for the varying cardiac demand during profound circulatory changes. *In-vitro* and in-silico results demonstrated similar trends to the simulated changes in patient state however the magnitude of hemodynamic changes were different, thus justifying the progression to *in-vitro* evaluation.

## Introduction

As patients implanted with rotary left ventricular assist devices (LVADs) progress through different activity levels throughout the day, under-pumping or over-pumping may occur which can lead to pulmonary congestion, impairment of right heart function and collapse of the left ventricle (LV) [1]. Due to the lower preload sensitivity of rotary LVADs in the conventional constant speed controller (CSC), when compared to the native heart [2], various physiological control techniques have been developed to match pump output to physiological requirements [3].

Starling and Visscher [4] demonstrated that LV contractility is proportional to LV end-diastolic pressure (PLVED) via the Starling mechanism [5]. Accordingly, many LVAD physiological control systems have attempted to duplicate the Starling mechanism while relying on pressure and flow sensors [6–11]. For example, Bullister el al. [12] proposed a physiological controller that maintained PLVED at a set point, thus increasing or decreasing cardiac output to prevent respective changes in PLVED. Although this method was superior to CSC, the level of resting PLVED alters significantly among individuals [13–15], while maintaining a fixed PLVED during various circulatory perturbations would also require excessive pump speed variations [16].

Recently, our group proposed a preload-based Starling-like controller (SLC) that could imitate the native heart’s preload sensitivity [16]. The relation between pump flow output and preload was sigmoid-like and could be formulated using a third-order polynomial equation [16]. This non-linear relationship between pump flow and ventricular preload gave the controller the ability to make large adjustments in pump flow at low preloads in order to avoid ventricular suction, while reducing pump power at high preloads to avoid over pumping. Using a numerical model of the cardiovascular system, it was demonstrated that a single control line SLC outperformed a constant pulsatility ratio controller [8] and CSC. SLC resulted in higher mean pump flow ($\bar{{\text{Q}}_{\text{P}}}$) during exercise simulations, and prevented ventricular suction while maintaining suitable hemodynamic parameters during simulations of blood loss and reduced LV contractility (LVC).

For any newly proposed physiological control system, there is a hierarchy of studies that can be performed to evaluate its efficacy, each having its own advantages and disadvantages. While numerical models are commonly used as the first step during controller evaluation due to the simplicity of model set-up and high reproducibility in results, it is difficult to model the rotary blood pump dynamics accurately due to its complexity. As a result, simulation results involving transient changes of pump speed to a particular perturbation or control action as well as pump flow or speed pulsatility are less reliable. Evaluation of the control techniques in a mock circulation loop (MCL) allows for real world use of an actual pump and therefore a more accurate representation of the pump speed response to simulated changes in patient state. Additionally, flow and pressure sensors can be used as feedback to the controller, which is a more realistic situation than would be obtained using a numerical model. As the steady-state response of the SLC system has only been evaluated in-silico, further assessment using a more advanced bench top apparatus is required to observe the dynamic effects of the pump and other circulatory compartments with this control method. It is therefore of interest to enhance our previous numerical evaluation of the SLC by comparing the SLC system with the clinically used CSC in a validated MCL. In this work, both the temporal and steady state responses of the SLC to three different test scenarios, including moderate exercise, blood-loss and a major reduction in LVC, was assessed and compared with CSC.

## Methodology

### Description of the mock circulation loop

**Fig 1:**
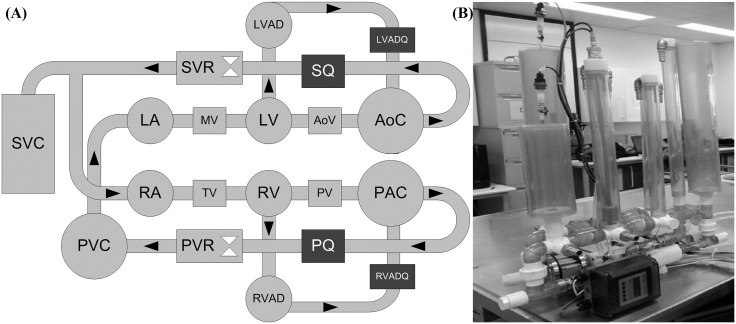
(A) Schematic of the dual circuit mock circulation loop, and (B) Photograph of the MCL. LA, left atrium; MV, mitral valve; LV, left ventricle; AoV, aortic valve; AoC, systemic arterial compliance; SQ, systemic flow meter; SVR, systemic venous resistance; SVC, systemic venous compliance; RA, right atrium; TV, tricuspid valve; RV, right ventricle; PV, pulmonary valve; PAC, pulmonary arterial compliance; PQ, pulmonary flow meter; PVR, pulmonary venous resistance; PVC pulmonary venous compliance; LVAD left ventricular assist device.

A physical MCL (Fig 1) including systemic and pulmonary circulations was used for this study [17]. Four independent Windkessel chambers were employed to represent the lumped systemic and pulmonary arterial and venous compliance. The systemic and pulmonary vascular resistances were manipulated by socket valves (VMP025.03X.71, AKO Alb. Klein Ohio LLC, USA). A series of electro-pneumatic regulators (ITV2030-012BS5, SMC Pneumatics, Tokyo, Japan) and 3/2 way solenoid valves (VT325-035DLS, SMC Pneumatics, Tokyo, Japan) were used to control ventricular systole (i.e. contractility, heart rate and systolic interval) and passively fill the heart chambers. A Starling mechanism was implemented for both the left and right ventricles to actively control the ventricular pressure through the electro-pneumatic regulator current supply based on ventricular preload[18]. The mitral, aortic, tricuspid and pulmonary valves were simulated using mechanical check valves. In this study, a mixture of water and glycerol (60% water/40% glycerol by mass) was used as the working fluid to deliver asymptotic viscosity (3.5 mPa.s) and density (1100 kg/m^3^) similar to that of blood at 37°C.

A VentrAssist LVAD (formally of Ventracor Ltd., Sydney, Australia) was used to support the simulated failing heart in the MCL. The LVAD was cannulated with inflow connected to the LV and outflow to the aorta. The left and right atrial, left and right ventricular, systemic arterial, pulmonary arterial and LVAD inlet/outlet pressures were measured using silicone-based transducers (PX181B-015C5V, Omega Engineering, Stamford, CT, USA). Systemic and pulmonary flow rates were recorded using magnetic flow meters (IFC010, KROHNE, Duisburg, Germany) while LVAD flow rate was monitored by an ultrasonic flowmeter (TS410-10PXL, Transonic Systems, NY, USA). All data were sampled at 2 KHz and recorded using a dSPACE 1103 (dSPACE, Wixom, MI, USA). The MCL operational and control software were developed in MATLAB/SIMULINK (The MathWorks, Natick, MA).

### Preload-based Starling-like controller

The immediate response of the SLC was formulated as a sigmoid relationship between LV stroke work and mean PLVED (PLVED_m_) [19] to emulate the Starling mechanism of the native heart. A control line was generated by a third-order polynomial function (Eq 1) fitted to Guyton’s data [19]. This line relates the desired mean pump flow ($\bar{{\text{Q}}_{{\text{P}}_{\text{Ref}}}}$) to PLVED_m_. In cases when the lower preload sensitivity at high flows (compared with linear preload control) turned out to be insufficient for any given patient, the preload sensitivity of the curve as a whole can be increased. A scaling factor (K) was added to provide a means of altering the pump sensitivity to changes in PLVED_m_, which makes Eq 1 adaptive with different preload sensitivities of different patients [16].

$$\bar{{\text{Q}}_{{\text{P}}_{\text{Ref}}}}=(0.0003{*\text{PLVED}}_{\text{m}}^{3}-0.0276{*\text{PLVED}}_{\text{m}}^{2}+0.9315{*\text{PLVED}}_{\text{m}}-0.0928)*\text{K}$$ (1)

where PLVED_m_ represents the mean LV end-diastolic pressure. As the heart beat timing was managed by the automatic MCL controller, the time of PLVED could be precisely determined. By sampling the LV pressure at this moment, PLVED was acquired. The measured PLVED was then passed through a low pass digital filter of 0.25 Hz to smooth the variation and acquire the mean PLVED.

**Fig 2:**
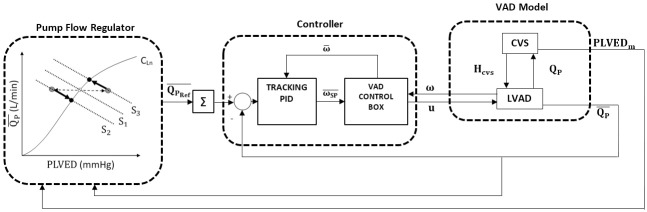
Block diagram of the control system. CVS, cardiovascular system; VAD, ventricular assist device, LVAD, left VAD; u, mean pulse-width modulation drive signal to the LVAD; H_CVS_, differential pressure between the left ventricle and the aorta; C_Ln_, Control line; S_1_, original state; S_2_ and S_3_, deviated states; Q_P_, pump flow; $\bar{{\text{Q}}_{\text{P}}}$, mean pump flow; $\bar{{\text{Q}}_{{\text{P}}_{\text{Ref}}}}$, The reference (desired) mean pump flow; PLVED_m_, mean left ventricular end diastolic pressure; ω, pump speed; $\bar{\omega }$, mean pump speed; ${\bar{\omega }}_{{}_{\text{SP}}}$, mean pump speed set-point; Grey circles, position of operating points after changes in states; Black circles, position of operating points upon arriving at the new steady state located at the intersection between the control line and the new system line. The controller drives the changes in the operating points along the path indicated by the arrows along the new system line; PLVED_m_ serves as the input to the preload controller (Eq 1); ∑, 1-second moving average filter.

Any change in state (Fig 2) caused a deviation in the operating point from its original position on the control line to other system lines. The controller then forced the operating point back to the control line along a linear path, which conformed closely to the trajectories of the linearized system lines. Changes in state were thus countered by moving the operating point up or down the control line. The pump speed was controlled to maintain the operating point at its intersection between the control line and the system line.

Fig 2 also depicts a nonlinear relationship between the pump flow and the ventricular preload. This non-linearity feature is one of the most important characteristics of the SLC; it gave the controller the ability to deliver a very high preload sensitivity at low preloads (to avoid ventricular suction) whilst a flat slope at high preloads ensured the avoidance of over-pumping. This was in contrast to linear versions of preload control, such as Stevens et al. [9], which should not be considered as true Starling-like control. A more detailed description of the SLC implementation can be found in [16].

### Controller implementation

Pump speed was measured based on the back electromotive force of the VentrAssist motor coils. A proportional—integral—derivative (PID) controller was developed to track the desired pump flow, $\bar{{\text{Q}}_{\text{P}}{}_{\text{Ref}}}$, by adjusting the average pump speed. Eq (2) defines the tracking PID transfer function, automatically discretized by MATLAB/SIMULINK using a sampling period of 0.0005 s.

$$\{\begin{array}{c} \text{PID}\left(\text{S}\right)=\left({\text{K}}_{\text{P}}+\frac{{\text{K}}_{\text{I}}}{\text{S}}+{\text{K}}_{\text{D}}\text{S}\right)e\left(\text{S}\right)+\bar{\omega }(\text{S})\\ \text{e}\left(\text{S}\right)=\;\bar{{\text{Q}}_{{\text{P}}_{\text{Ref}}}}\left(\text{S}\right)-\bar{{\text{Q}}_{\text{P}}}(\text{S}) \end{array}$$ (2)

where $\bar{\omega }$ stands for mean pump rotational speed and S is the complex frequency.

The PID gains were tuned based on Ziegler–Nichols method [20] to achieve a 5% settling time of 10 s and a 10% maximum overshoot of the final value, in response to a step change in the mean $\bar{{\text{Q}}_{\text{P}}}$ set point from 1.80 L/min (corresponding to a pump speed of 1800 rpm, i.e. the minimum operational speed) to the baseline value of 5.2 L/min. The resultant PID controller gains, K_P_, K_I_ and K_D_ were set to 130 rpm.min/L, 162.5 rpm.min/L/s, and 58.5 rpm.s.min/L, respectively, which provided settling and response times of 2.0 s and 4.5 s, with no overshoot.

Pump speed $\bar{{\text{Q}}_{\text{P}}}$, and PLVED feedback signals were passed through a first-order transfer function to obtain their mean values. The cutoff frequency of the filter was empirically set to 0.25 Hz to abate the short-term variability of the feedback signals without compromising the system bandwidth. To minimize the tracking signal noise, a moving average filter with cutoff frequency of 0.5 Hz was placed after $\bar{{\text{Q}}_{{\text{P}}_{\text{Ref}}}}$ but before the controller.

### Experimental protocol

Both CSC and SLC were subjected to the same assessment protocol. The scaling factor (K) was set to 1.0 for the SLC, while the corresponding speed of the CSC was set to 2100 rpm. Each experiment started with a baseline LV failure condition at rest for 120 s to allow the system to settle prior to performing a step change to one of the three test scenarios (i.e. exercise, blood loss and reduced LVC). Upon transitioning to the new states, the experiments were continued for another 120 s to achieve the post-transition steady state.

To simulate an exercise scenario, 700 mL of fluid was shifted from the systemic venous compliance (SVC) chamber into the circulation, emulating the action of the muscle pump in increasing venous return. Heart rate was increased from 60 to 90 bpm, while LVC was increased from a dP/dt_max_ of 1040 to 1880 mmHg/s[21]. Systemic vascular resistance (SVR) and pulmonary vascular resistance (PVR) were decreased from 1300 to 600 dyne.s.cm^-5^ and from 110 to 40 dyne.s.cm^-5^ respectively.

To simulate blood loss, 300 mL fluid was shifted from the circulation into the systemic venous compliance chamber to emulate blood flowing into the legs. SVR and PVR were increased from 1300 to 1635 dyne.s.cm^-5^ and from 110 to 210 dyne.s.cm^-5^ respectively, to simulate vasoconstriction.

A major reduction in LVC was simulated by eliminating the LV contractility using the LV electro-pneumatic regulators. For all simulations, the change of parameters was immediate and simultaneous; however, the fluid shifts were completed over a longer duration (but no more than 20 seconds) due to the MCL dynamics. In this MCL, fluid shifts from the SVC to the heart and vice versa were controlled by adjusting the air pressure in the SVC chamber using a manual ball valve. The key MCL parameters used to mimic baseline, exercise, blood-loss, and reduced LVC conditions are listed in Table 1.

**Table 1:** Key MCL parameters for mimicking different hemodynamic conditions.

Variable	Baseline	Exercise	Blood-loss	LVC Reduction
Heart rate (bpm)	60	90	65	60
SVR (Dynes.s/cm^5^)	1300	600	1635	1300
PVR (Dynes.s/cm^5^)	110	40	210	110
Circulation fluid shift	---	SVC → RV (700mL)	RV → SVC (300mL)	---
C_lv_ (mmHg/s)	1040	1880	1040	25

PVR, pulmonary vascular resistance; SVR, systemic vascular resistance; RV, right ventricle; SVC, systemic venous compliance chamber; LV, left ventricle; LVC, LV contractility; C_lv_, LV end systolic elastane.

### Performance evaluation

The performance of SLC and CSC was compared by observing the changes in $\bar{{\text{Q}}_{\text{P}}}$, mean total cardiac output ($\bar{\text{CO}}$), mean arterial pressure (MAP) and left atrial pressure ($\bar{{\text{P}}_{\text{la}}}$) while transitioning from the baseline state to exercise, blood-loss, and reduced LVC scenarios. In this study, a performance metric to provide a quantitative comparison aspect of clinical context with each controller was employed. Accordingly, the average deviation per second of the MAP, PLVED, and $\bar{\text{CO}}$ from the respective predefined physiological limits (Table 2), that denoted the root mean square hemodynamic deviation (RMSHD), was determined[22, 23]. A lower RMSHD promises a better clinical performance of the controller [22].

**Table 2:** Upper and lower limits for the key hemodynamic variables.

Variable (unit)	Lower Limit	Upper Limit
Mean arterial pressure (mmHg)	80	120
Left ventricular end diastolic pressure (mmHg)	2	15
Cardiac output: Rest and hemorrhage (L/min)	4	6
Cardiac output: Exercise (L/min)	6	11

Deviations outside of these bounds were recorded and used to compare the physiological performance of the control systems.

To calculate RMSHD, let y_x_ stand for any of the three previously mentioned hemodynamic variables (i.e. MAP, PLVED, $\bar{\text{CO}}$), and let LL_x_ and UL_x_ define the lower and upper limits of the safe operating bound for that variable, respectively. The normalized square deviation (NSD_x_(t)) of y_x_(t) outside of LL_x_ and UL_x_ is calculated as follows:

$${\text{NSD}}_{\text{x}}\left(\text{t}\right)={\left(\frac{{\text{v}}_{\text{x}}\left(\text{t}\right)}{\frac{{\text{LL}}_{\text{x}}+{\text{UL}}_{\text{x}}}{2}}\right)}^{2}$$ (3)

where

$${\text{v}}_{\text{x}}\left(\text{t}\right)=\{\begin{array}{c} {\text{y}}_{\text{x}}\left(\text{t}\right)-{\text{UL}}_{\text{x}};{\text{ y}}_{\text{x}}\left(\text{t}\right)>U{\text{L}}_{\text{x}}\\ 0;{\text{LL}}_{\text{x}}<{\text{y}}_{\text{x}}\left(\text{t}\right)<U{\text{L}}_{\text{x}}\\ {\text{LL}}_{\text{x}}-{\text{y}}_{\text{x}}\left(\text{t}\right);{\text{ y}}_{\text{x}}\left(\text{t}\right)<L{\text{L}}_{\text{x}} \end{array}$$ (4)

Eq (5) formulates the squared hemodynamic deviation (SHD) of the variable y_x_:

$${\text{SHD}}_{\text{x}}=\int_{0}^{{\text{T}}_{\text{d}}}{\text{NSD}}_{\text{x}}(\text{t})$$ (5)

Finally, RMSHD is calculated as Eq (6):

$$\text{RMSHD}=\left(\frac{1}{{\text{T}}_{\text{d}}}\right)\sqrt{\left({\text{SHD}}_{{\text{P}}_{\text{ao}}}^{2}+{\text{SHD}}_{\text{PLVED}}^{2}+{\text{SHD}}_{\text{CO}}^{2}\right)}$$ (6)

## Results

Results were obtained to compare the performance of SLC and CSC systems under various hemodynamic perturbations. During the exercise simulation, starting hemodynamics were similar between SLC and CSC systems. At the onset of exercise, a drop in SVR and PVR resulted in an initial fall in systemic arterial pressure (P_SA_) and a rise in pump flow (Q_P_) despite an increase in heart rate with both controllers (Table 3). After a few seconds, fluid was shifted from the systemic venous compliance into the RV, which subsequently activated the ventricular Starling mechanism, causing both left and right ventricular contractility to increase. Consequently, P_SA_ and Q_P_ increased gradually before settling to a level higher than their baseline values with SLC and CSC.

**Table 3:** In-silico and *in-vitro* hemodynamic data at baseline (rest) and exercise for constant speed mode and Starling-like control.

Variable	Unit	In-Silico ^1^						*In-Vitro*					
		Constant Speed			Starling-like			Constant Speed			Starling-like		
		Baseline	Exercise	% change	Baseline	Exercise	% change	Baseline	Exercise	% change	Baseline	Exercise	% change
$\bar{\omega }$	rpm	2600	2600	0.0	2600	2980	14.6	2100±13	2100±15	0.0	2103±18	2295±18	9.1
MAP	mmHg	103	101	-1.9	103	104	1.0	89±0.2	95±0.3	6.7	89±0.3	96±0.2	7.9
$\bar{{\text{P}}_{\text{LA}}}$	mmHg	9	21	133.3	9	19	111.1	10.9±0.1	16.4±0.1	50.4	10.8±0.1	14.2±0.1	31.5
PLVED_m_	mmHg	7.4	18.9	155.4	7.4	15.6	110.8	9.5±0.1	15.3±0.2	61.1	9.3±0.1	13.8±0.4	48.4
$\bar{\text{CO}}$	L/min	5.6	8.9	58.9	5.6	9.2	64.3	5.2±0.1	10.1±0.1	94.2	5.2±0.1	10.4±0.1	100.0
$\bar{{\text{Q}}_{\text{P}}}$	L/min	5.6	6.7	19.6	5.6	8.5	51.8	5.2±0.1	6.2±0.1	19.2	5.2±0.1	7.1±0.2	36.5
RMSHD	%	0.0	11.2	---	0.0	0.6	---	0.0	26.4	---	0.0	12.7	---

$\bar{\omega }$, mean pump speed; MAP, mean arterial pressure; $\bar{{\text{P}}_{\text{LA}}}$, mean left atrial pressure; PLVED_m_, mean left ventricular end diastolic pressure; $\bar{\text{CO}}$, mean cardiac output; Q_P_, pump flow; $\bar{{\text{Q}}_{\text{P}}}$, mean pump flow; RMSHD, root mean squared hemodynamic deviation.

^1^ Results in the in-silico section were extracted from [16].

**Fig 3:**
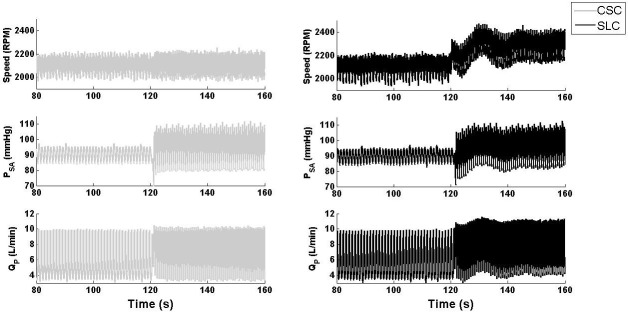
Transient response of LVAD speed, systemic pressure, and pump flow during a simulated transition from baseline to exercise. SLC, Starling-like control; CSC, constant speed control; P_SA_, systemic arterial pressure; Q_P_, pump flow.

**Fig 4:**
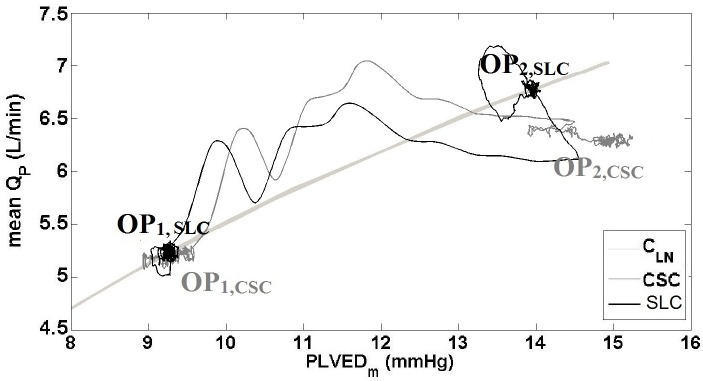
The relationship between mean pump flow and mean PLVED while transitioning from baseline to exercise, for the constant speed mode and Starling-like control. SLC, Starling-like control; CSC, constant speed control; Q_P_, pump flow; C_Ln_, control line (1); OP_1_, initial operational point; OP_2_, final operational point.

Fig 3 clearly showed that both the SLC and CSC demonstrated similar hemodynamic transition patterns; however, the magnitude of hemodynamic changes was greater with the SLC, especially for $\bar{{\text{Q}}_{\text{P}}}$ (Table 3). This was due to the increased pump speed with the SLC (from 2103 to 2295 RPM) compared to that of the CSC (constant at 2100 RPM), with the Starling-like relationship of the SLC during the transition to exercise shown in Fig 4. The improved performance of the SLC can be characterized by observing the final steady-state results shown in Table 3, which showed increased $\bar{{\text{Q}}_{\text{P}}}$ with the SLC (7.1 L/min) compared to CSC (6.2 L/min) after the exercise condition had settled. However, it should be noted that total $\bar{\text{CO}}$ was similar between the two controllers, and may indicate increased ventricular work with CSC compared to SLC. PLVED_m_ was decreased with the SLC (13.8 mmHg) compared to CSC (15.1 mmHg) during exercise, which also indicates reduced load on the LV. Moreover, the value of RMSHD for the SLC was 12.7%, less than the half of the value achieved when utilizing CSC (RMSH of 26.4%), indicating a significantly better clinical performance for the SLC.

When comparing *in-vitro* and in-silico results (Table 3), it was inferred that the trend in all hemodynamic variables remained the same when comparing SLC and CSC; however, the magnitude of changes were different due to the different platform settings and starting hemodynamics and pump speeds. In the numerical model the speed changes for SLC were higher (14.6%) when compared to *in-vitro* testing (9.1%), which resulted in higher $\bar{{\text{Q}}_{\text{P}}}$ changes in-silico. Furthermore, cardiac output increased in-silico and *in-vitro* to similar levels (by 5.4% and 5.8% respectively) with SLC, indicating a reduction in ventricular work with SLC. MAP changes were higher *in-vitro* for both CSC and SLC (6.7% and 7.9% increase respectively) when compared to the numerical model (1.9% decrease with CSC and 1% increase with SLC).

**Fig 5:**
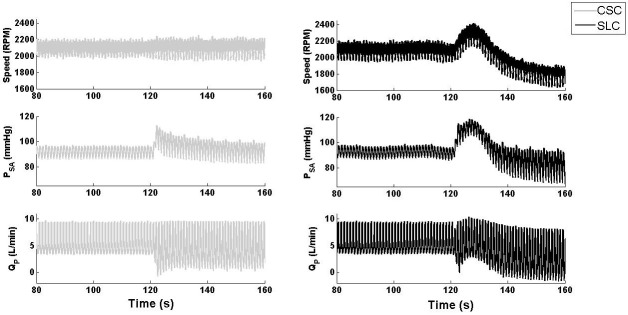
Transient response of LVAD speed, systemic pressure, and pump flow during a simulated transition from baseline to blood-loss. SLC, Starling-like control; CSC, constant speed control; P_SA_, systemic arterial pressure; Q_P_, pump flow.

**Fig 6:**
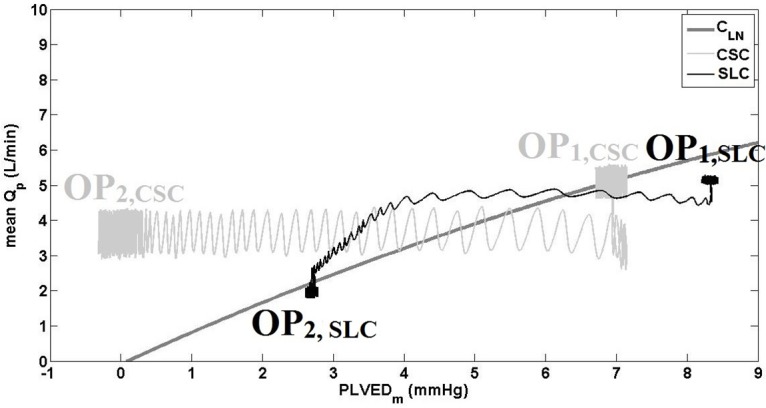
The relationship between mean pump flow and mean PLVED while transitioning from baseline to blood-loss, for the constant speed mode and Starling-like control. SLC, Starling-like control; CSC, constant speed control; Q_P_, pump flow; C_Ln_, control line (1); OP_1_, initial operational point; OP_2_, final operational point.

During the blood loss simulation, SVR and PVR were increased which resulted in increased MAP and a reduction in $\bar{{\text{Q}}_{\text{P}}}$ (Fig 5 and Table 4) with both CSC and SLC. As the process included a gradual shift in fluid over approximately 20 s, it can be assumed that the circulation volume was almost constant during the first few seconds of the transition. As the fluid shifted from the circulation to the SVC chamber, the mean circulatory filling pressure, systemic arterial pressure and $\bar{{\text{Q}}_{\text{P}}}$ all decreased with the SLC and CSC. However, the magnitude of hemodynamic changes varied greatly between systems. For instance, after the blood loss was completed, $\bar{{\text{Q}}_{\text{P}}}$ only decreased from 5.1 to 3.6 L/min with CSC which resulted in PLVED_m_ of -0.1 mmHg, indicating LV suction (Table 4). In contrast, the SLC reduced mean pump speed from 2096 to 1793 RPM, which reduced $\bar{{\text{Q}}_{\text{P}}}$ from 5.1 to 2.1 L/min and maintained PLVED_m_ above 2 mmHg, thus avoiding LV suction. This is evident in the relationship between $\bar{{\text{Q}}_{\text{P}}}$ and PLVED_m_ in Fig 6. Thus, it can be concluded that the SLC was able to reduce $\bar{{\text{Q}}_{\text{P}}}$ adequately to maintain an adequate safety margin against LV suction while the CSC could not. Meanwhile, RMSHD was 199% for CSC, showing a dramatic reduction in performance compare with the SLC system (RMSHD value of 58%).

**Table 4:** In-silico and *in-vitro* hemodynamic data at baseline (rest) and blood-loss for constant speed mode and Starling-like control.

Variable	Unit	In-Silico ^1^						*In-Vitro*					
		Constant Speed			Starling-like			Constant Speed			Starling-like		
		Baseline	Blood loss	% change	Baseline	Blood loss	% change	Baseline	Blood loss	% change	Baseline	Blood loss	% change
$\bar{\omega }$	rpm	2600	2600	0.0	2600	2115	-18.7	2100±13	2100±20	0.0	2096±19	1793±25	-14.6
MAP	mmHg	103	96	-6.8	103	84	-18.4	91±0.2	92±0.2	1.1	92±0.3	81±0.3	-13.6
$\bar{{\text{P}}_{\text{LA}}}$	mmHg	9	4	-55.5	9	5.4	-40.0	9.2±0.1	1.4±0.0	-84.8	10.0±0.1	3.2±0.1	-68.0
PLVED_m_	mmHg	7.4	-0.4	-105.4	7.4	4.9	-33.8	7.1±0.1	-0.1±0.2	-101.4	8.3±0.1	2.7±0.1	-67.5
$\bar{\text{CO}}$	L/min	5.6	5.3	-5.4	5.6	4.4	-21.4	5.2±0.1	3.9±0.1	-25.0	5.3±0.1	3.7±0.1	-30.2
$\bar{{\text{Q}}_{\text{P}}}$	L/min	5.6	5.3	-5.4	5.6	3.7	-33.9	5.1±0.1	3.6±0.2	-29.4	5.1±0.1	2.1±0.1	-58.8
RMSHD	%	0.0	11.2	---	0.0	0.6	---	0.0	26.4	---	0.0	12.7	---

$\bar{\omega }$, mean pump speed; MAP, mean arterial pressure; $\bar{{\text{P}}_{\text{LA}}}$, mean left atrial pressure; PLVED_m_, mean left ventricular end diastolic pressure; $\bar{\text{CO}}$, mean cardiac output; Q_P_, pump flow; $\bar{{\text{Q}}_{\text{P}}}$, mean pump flow; RMSHD, root mean squared hemodynamic deviation.

^1^ Results in the in-silico section were extracted from [16].

Similar to the exercise scenario, *in-vitro* results showed similar trends when compared to the in-silico results for both CSC and SLC (Table 4). Although the magnitude of changes in hemodynamics in response to blood loss were different between *in-vitro* and in-silico simulations, the differences between CSC and SLC in terms of percentage changes were comparable. The SLC decreased pump speed by 18.7% and 14.6% when evaluated in-silico and *in-vitro* respectively. Differences in $\bar{{\text{Q}}_{\text{P}}}$ changes between CSC and SLC were similar both in-silico and *in-vitro* with the SLC decreasing $\bar{{\text{Q}}_{\text{P}}}$ by 28.5% and 29.4% respectively.

**Fig 7:**
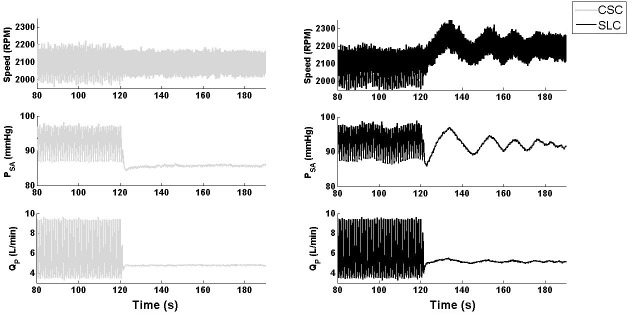
Transient response of LVAD speed, systemic pressure, and pump flow during a simulated transition from baseline to reduced left ventricular contractility. SLC, Starling-like control; CSC, constant speed control; P_SA_, systemic arterial pressure; Q_P_, pump flow.

**Fig 8:**
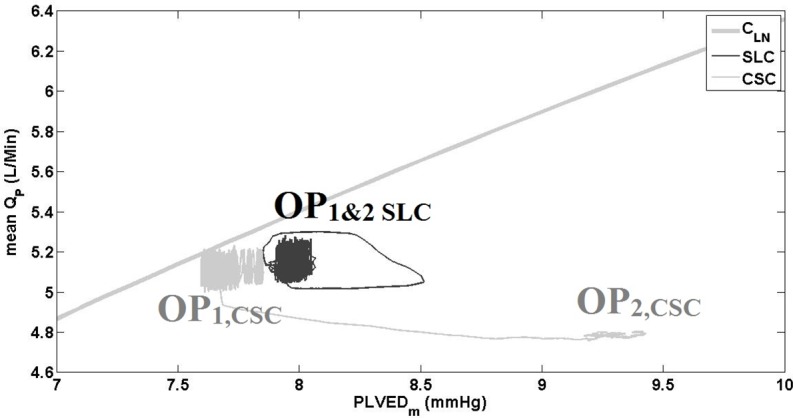
The relationship between mean pump flow and mean PLVED while transitioning from baseline to reduced left ventricular contractility, for the constant speed mode and Starling-like control. SLC, Starling-like control; CSC, constant speed control; Q_P_, pump flow; C_Ln_, control line (1); OP_1_, initial operational point; OP_2_, final operational point.

During a major reduction in LVC with CSC, $\bar{{\text{Q}}_{\text{P}}}$ decreased from 5.1 to 4.8 L/min (Figs 7 and 8). Although this was within our clinically acceptable ranges, the SLC was able to maintain $\bar{{\text{Q}}_{\text{P}}}$ at 5.1 L/min through an increase in pump speed from 2096 to 2210 RPM (Table 5). However, the SLC appeared to have an increased settling time during the major reduction in LVC compared to other simulations (Fig 7), which can be attributed to the elimination of the flow-balancing ventricular Starling response, along with the dynamics of the systemic and pulmonary circulations connected in series.

**Table 5:** In-silico and *in-vitro* hemodynamic data at baseline (rest) and reduced LV contractility scenario (LVC) for constant speed mode and Starling-like control.

Variable	Unit	In-Silico ^1^						*In-Vitro*					
		Constant Speed			Starling-like			Constant Speed			Starling-like		
		Baseline	LVC	% change	Baseline	LVC	% change	Baseline	LVC	% change	Baseline	LVC	% change
$\bar{\omega }$	rpm	2600	2600	0.0	2600	2700	3.8	2100±13	2100±1	0.0	2096±17	2210±10	5.4
MAP	mmHg	103	97	-5.8	103	103	0.0	92±0.1	86±0.1	-6.5	92±0.3	92±0.2	0.0
$\bar{{\text{P}}_{\text{LA}}}$	mmHg	9	12	33.3	9	9	0.0	9.2±0.1	9.8±0.1	6.5	9.7±0.1	8.6±0.1	-11.3
PLVED_m_	mmHg	7.4	11	48.6	7.4	7.4	0.0	7.7±0.1	9.2±0.1	19.5	8.0±0.1	7.9±0.1	-1.3
$\bar{\text{CO}}$	L/min	5.6	5.2	-7.1	5.6	5.6	0.0	5.2±0.1	4.8±0.1	-7.7	5.3±0.1	5.2±0.1	-1.9
$\bar{{\text{Q}}_{\text{P}}}$	L/min	5.6	5.2	-7.1	5.6	5.6	0.0	5.1±0.1	4.8±0.1	-5.9	5.1±0.1	5.1±0.1	0.0
RMSHD	%	0.0	0.0	---	0.0	0.0	---	0.0	0.0	---	0.0	0.0	---

$\bar{\omega }$, mean pump speed; MAP, mean arterial pressure; $\bar{{\text{P}}_{\text{LA}}}$, mean left atrial pressure; PLVED_m_, mean left ventricular end diastolic pressure; $\bar{\text{CO}}$, mean cardiac output; Q_P_, pump flow; $\bar{{\text{Q}}_{\text{P}}}$, mean pump flow; RMSHD, root mean squared hemodynamic deviation.

^1^ Results in the in-silico section were extracted from [16].

Similar to the previous experiments the CSC and SLC exhibited similar responses to a transition from baseline to LVC reduction when comparing in-silico and *in-vitro* results (Table 5). The SLC maintained hemodynamic parameters during an LVC reduction by increasing pump speed by 3.8% (in-silico) and 5.4% (*in-vitro*), which subsequently reduced LV preload when compared to CSC.

## Discussion

To date, various physiologically responsive controllers have been proposed for LVAD support [3] to cater for the varying metabolic demand of the pump-assisted patients while undergoing different activities in their daily lives. Gaddum et al (2014) and Schima et al (2006) utilized pulsatile-based controllers to imitate the native Starling flow sensitivity [7, 10]. Although their results demonstrated the strength of pulsatility control over CSC, there are several limitations associated with their controllers. The main issue with the proposed pulsatility controller was that pump pulsatility (flow, current, pressure gradient, or speed) is a consequence of LV contraction, which is dependent on LV preload. In cases of severe LV failure, the LV does not possess sufficient capacity to influence pulsatility and thus the dynamic range of pulsatility indexes is small. More importantly, pulsatility control is not feasible in cases with zero LV contractility. In addition, our published numerical studies demonstrated the limitations of those pulsatility index control strategies during exercise, blood loss, and left ventricular contractility reduction that agree with the clinical studies [16, 24]. Such limitations did not exist with our SLC system, as demonstrated by the capacity to restore hemodynamics even with complete elimination of LVC (and thus pulsatility).

A Starling-like LVAD control method was developed by Stevens et al. [9] in 2011, which used a conventional PID (proportional-integral-derivative) technique to regulate pump flow as a function of left atrial pressure. The Starling mechanism was approximated with a linear relation between average left atrial pressure ($\bar{{\text{P}}_{\text{LA}}}$) and mean pump flow ($\bar{{\text{Q}}_{\text{P}}}$) where the gradient between these two variables (slope of the line) alters to respond to different physiological conditions. However, adding an automatic sensitivity regulator necessitated an extra PI controller cascaded with the original PID that increased the system complexity and increased the cost of gain tuning. On the contrary, our SLC demonstrated similar suction prevention and exercise capacity to those presented by Stevens et al without the requirement of an additional PI controller cascade. Furthermore, Stevens’ model did not include a flat slope at higher preload values, which is crucial for Starling-like controllers to avoid over-pumping at high preloads.

Compared to CSC, the SLC evaluated in this study was able to synchronize systemic and pulmonary flow rates irrespective of variations in venous return by emulating the Starling mechanism of the native heart. The SLC produced a lower PLVED than CSC during exercise and reduced LV contractility scenarios, thus potentially providing improved ventricular unloading. Meanwhile, there is evidence that LV suction under CSC may cause a significant reduction in right ventricular performance through endocardial damage and septal shift [8, 25]. Improved flow-balancing with the SLC may reduce the incidence of pulmonary congestion; an incident that may otherwise lead to long-term right ventricular failure [1]. Meanwhile, maintaining adequate LV preload with the SLC will also prevent intermittent LVAD flow stoppages and ventricular arrhythmias associated with left ventricular suction, even in severe blood loss conditions as simulated in this study.

When simulating a major reduction in LVC, we observed increased preload with CSC whilst MAP dropped. The SLC responded to such rise in preload by increasing the pump speed and flow, which subsequently returned the preload to its previous value. The results showed little benefit of the SLC over constant speed mode, evident by zero RMSHD value for the both methods. Under the SLC, both $\bar{{\text{Q}}_{\text{P}}}$ and MAP were maintained at 5.1 L/min and 92 mmHg respectively, whilst we saw a decrease in mean pump flow to 4.8 L/min with MAP at 86 mmHg for the fixed speed operational mode. The results showed CSC is in fact suitable to tolerate LVC reduction, while the SLC only provides minimal benefits. Meanwhile, the long settling time with the SLC during LVC reduction could be considered a limitation with our controller; however, no adverse events (ventricular suction, venous congestion) occurred.

Results in the present study clearly established similar trends between in-silico and *in-vitro* testing [16], with the SLC demonstrating superior performance when compared to CSC in all scenarios in both evaluation platforms. The absence of the autonomic baroreflex mechanism in the MCL resulted in a smaller increase in mean arterial and left atrial pressures compared to the numerical simulation; however differences between the two platforms were minor. During the blood loss simulation, large spikes in pump flow indicating LV suction occurred with CSC, which was in agreement with our previously published numerical study [16]. Although in-silico and *in-vitro* results showed similar trends during changes in patient state, there were differences in the magnitude of changes, which could be attributed to differences in simulated patient hemodynamics, starting pump speeds, the lack of a baroreflex *in-vitro*, and the real-world use of a pump and sensors *in-vitro*. Providing an exact replica between both simulation platforms is difficult, and the focus of this evaluation paper was to demonstrate the feasibility of our SLC to prevent adverse events under various simulated patient states in an MCL. A full comparison between the two testing benchtops, although interesting, is beyond the scope of this *in-vitro* physiological control work.

Although this study focused on using a single preload control line, a full controller should be capable of adapting to variations in the left ventricle (LV) function by automatically adjusting the scaling factor. The process of adapting curves and changing the scaling factor was initially introduced by Salamonsen et al (2012)[8] and Gaddum et al (2014)[7]. At the time of LVAD implantation, adjustments to different Starling curves ensures optimal LV unloading for each specific patient with the clinician input to match the initial control line with each individual. After LVAD implantation, changes in the preload sensitivity (i.e. scaling factor) of the control line might still be necessary to adapt to longer term changes, such as with the progression of the LV disease.

## Conclusion

Our *in-vitro* study clearly established the superiority of the preload-based SLC over CSC while transitioning from the baseline state to exercise, blood loss, and a major reduction in LVC. The SLC was able to provide a greater pump flow and cardiac output during exercise as compared to the conventional CSC, with less loading on the heart. In addition, it maintained a better safety margin against LV suction during blood loss. Although the CSC maintained suitable hemodynamics during a reduction in LVC, the SLC maintained almost perfect hemodynamic stability. The SLC has the potential to improve patient outcomes substantially through improved cardiac preservation and increased responsiveness to the patient’s requirements in the multiple physiological conditions faced by the LVAD patient—both in hospital and at home.

## References

[1] Haddad F, Doyle R, Murphy DJ, Hunt SA. Right ventricular function in cardiovascular disease, part ii pathophysiology, clinical importance, and management of right ventricular failure. Circulation. 2008;117(13):1717–31. 10.1161/CIRCULATIONAHA.107.653584 18378625

[2] Salamonsen RF, Mason DG, Ayre PJ. Response of rotary blood pumps to changes in preload and afterload at a fixed speed setting are unphysiological when compared with the natural heart. Artificial Organs. 2011;35(3):E47–E53. 10.1111/j.1525-1594.2010.01168.x 21355872

[3] AlOmari A-HH, Savkin AV, Stevens M, Mason DG, Timms DL, Salamonsen RF, et al Developments in control systems for rotary left ventricular assist devices for heart failure patients: A review. Physiological measurement. 2013;34(1):R1 10.1088/0967-3334/34/1/R1 23242235

[4] Starling E, Visscher M. The regulation of the energy output of the heart. The Journal of physiology. 1927;62(3):243–61. 16993846 10.1113/jphysiol.1927.sp002355 PMC1514842

[5] Guyton AC. Circulatory physiology: Cardiac output and its regulation. The American Journal of the Medical Sciences. 1965;219(1):122.

[6] Bakouri MA, Salamonsen RF, Savkin AV, AlOmari AHH, Lim E, Lovell NH. A sliding mode-based Starling-Like controller for implantable rotary blood pumps. Artificial Organs. 2014;38(7):587–93. 10.1111/aor.12223 24274084

[7] Gaddum NR, Stevens M, Lim E, Fraser J, Lovell N, Mason D, et al Starling-like flow control of a left ventricular assist device: In-vitro validation. Artificial Organs. 2014;38(3):E46–E56. 10.1111/aor.12221 24372519

[8] Salamonsen RF, Lim E, Gaddum N, AlOmari AHH, Gregory SD, Stevens M, et al Theoretical foundations of a Starling-like controller for rotary blood pumps. Artificial Organs. 2012;36(9):787–96. 10.1111/j.1525-1594.2012.01457.x 22626056

[9] Stevens MC, Gaddum NR, Pearcy M, Salamonsen RF, Timms DL, Mason DG, et al., editors. Frank-Starling control of a left ventricular assist device. Engineering in Medicine and Biology Society, EMBC, 2011 Annual International Conference of the IEEE; 2011: IEEE. 10.1109/IEMBS.2011.6090314 22254563

[10] Schima H, Vollkron M, Jantsch U, Crevenna R, Roethy W, Benkowski R, et al First clinical experience with an automatic control system for rotary blood pumps during ergometry and right-heart catheterization. The Journal of heart and lung transplantation. 2006;25(2):167–73. 10.1016/j.healun.2005.09.008 16446216

[11] Bullister E, Reich S, Sluetz J. Physiologic control algorithms for rotary blood pumps using pressure sensor input. Artificial Organs. 2002;26(11):931–8. 12406146 10.1046/j.1525-1594.2002.07126.x

[12] Bullister E, Reich S, d'Entremont P, Silverman N, Sluetz J. A Blood Pressure Sensor for Long-Term Implantation. Artificial Organs. 2002;25(5):376–9. 10.1046/j.1525-1594.2001.025005376.x 11403667

[13] Salamonsen RF, Pellegrino V, Fraser JF, Hayes K, Timms D, Lovell NH, et al Exercise studies in patients with rotary blood pumps: Cause, effects, and implications for starling-like control of changes in pump flow. Artificial Organs. 2013;37(8):695–703. 10.1111/aor.12070 23638682

[14] Jacquet L, Vancaenegem O, Pasquet A, Matte P, Poncelet A, Price J, et al Exercise capacity in patients supported with rotary blood pumps is improved by a spontaneous increase of pump flow at constant pump speed and by a rise in native cardiac output. Artificial Organs. 2011;35(7):682–90. 10.1111/j.1525-1594.2011.01227.x 21615428

[15] Mancini D, Goldsmith R, Levin H, Beniaminovitz A, Rose E, Catanese K, et al Comparison of exercise performance in patients with chronic severe heart failure versus left ventricular assist devices. Circulation. 1998;98(12):1178–83. 9743508 10.1161/01.cir.98.12.1178

[16] Mansouri M, Salamonsen RF, Lim E, Akmeliawati R, Lovell NH. Preload-based Starling-like control for rotary blood pumps: Numerical comparison with pulsatility control and constant speed operation. PLoS ONE. 2015;10(4):e0121413 10.1371/journal.pone.0121413 25849979 PMC4388698

[17] Timms DL, Gregory SD, Greatrex NA, Pearcy MJ, Fraser JF, Steinseifer U. A compact mock circulation loop for the in vitro testing of cardiovascular devices. Artificial Organs. 2011;35(4):384–91. 10.1111/j.1525-1594.2010.01088.x 20883450

[18] Gregory SD, Stevens M, Timms D, Pearcy M, editors. Replication of the Frank-Starling response in a mock circulation loop. Engineering in Medicine and Biology Society, EMBC, 2011 Annual International Conference of the IEEE; 2011: IEEE. 10.1109/IEMBS.2011.6091683 22255906

[19] Guyton AC. Circulatory physiology: Cardiac output and its regulation. Philadelphia and London: W.B. Saunders Company; 1963 p. 237–9.

[20] Zinober AS. Deterministic Nonlinear Control of Uncertain Systems: IET; 1990.

[21] Grossman W, Haynes F, Paraskos JA, Saltz S, Dalen JE, Dexter L. Alterations in preload and myocardial mechanics in the dog and in man. Circulation research. 1972;31(1):83–94. 5038740

[22] Mansouri M. Physiological control of an implantable rotary blood pump. Kuala Lumpur, Malaysia: University of Malaya; 2016.

[23] Stevens MC. Automatic control of dual LVADs as a BiVADs. Brisbane, QLD, AUSTRALIA: The University of Queensland; 2014.

[24] Lim E, Salamonsen RF, Mansouri M, Gaddum N, Mason DG, Timms DL, et al Hemodynamic response to exercise and head-up tilt of patients implanted with a rotary blood pump: A computational modeling study. Artificial Organs. 2015;39(2):E24–E35. 10.1111/aor.12370 25345482

[25] Salamonsen RF, Lim E, Moloney J, Lovell NH, Rosenfeldt FL. Anatomy and physiology of left ventricular suction induced by rotary blood pumps. Artificial Organs. 2015;39(8):681–90. 10.1111/aor.12550 26146861

